# fastSW: Efficient Piecewise Linear Approximation of Quaternion-Based Orientation Sensor Signals for Motion Capturing with Wearable IMUs

**DOI:** 10.3390/s21155180

**Published:** 2021-07-30

**Authors:** Florian Grützmacher, Jochen Kempfle, Kristof Van Laerhoven, Christian Haubelt

**Affiliations:** 1 Institute of Applied Microelectronics and Computer Engineering, University of Rostock, 18051 Rostock, Germany; christian.haubelt@uni-rostock.de; 2 Department of Electrical Engineering and Computer Science, University of Siegen, 57076 Siegen, Germany; jochen.kempfle@uni-siegen.de (J.K.); kvl@eti.uni-siegen.de (K.V.L.)

**Keywords:** piecewise linear approximation, segmentation, motion capturing, IMU, wearable sensors, orientation, quaternion

## Abstract

In the past decade, inertial measurement sensors have found their way into many wearable devices where they are used in a broad range of applications, including fitness tracking, step counting, navigation, activity recognition, or motion capturing. One of their key features that is widely used in motion capturing applications is their capability of estimating the orientation of the device and, thus, the orientation of the limb it is attached to. However, tracking a human’s motion at reasonable sampling rates comes with the drawback that a substantial amount of data needs to be transmitted between devices or to an end point where all device data is fused into the overall body pose. The communication typically happens wirelessly, which severely drains battery capacity and limits the use time. In this paper, we introduce fastSW, a novel piecewise linear approximation technique that efficiently reduces the amount of data required to be transmitted between devices. It takes advantage of the fact that, during motion, not all limbs are being moved at the same time or at the same speed, and only those devices need to transmit data that actually are being moved or that exceed a certain approximation error threshold. Our technique is efficient in computation time and memory utilization on embedded platforms, with a maximum of 210 instructions on an ARM Cortex-M4 microcontroller. Furthermore, in contrast to similar techniques, our algorithm does not affect the device orientation estimates to deviate from a unit quaternion. In our experiments on a publicly available dataset, our technique is able to compress the data to 10% of its original size, while achieving an average angular deviation of approximately 2° and a maximum angular deviation below 9°.

## 1. Introduction

Motion capturing is the process of estimating a human’s posture and movements over time using a computer controlled sensor system. Capturing can happen on the full body or only on specific parts, such as the fingers, the face, the upper or lower body limbs, or just a single arm. It not only has become an integral part of computer animation in entertainment businesses but is also of growing interest in many other fields, such as medical applications, such as gait analysis, in sports, in activity recognition, or, more recently, in virtual and augmented reality. Depending on the task, limb or joint positions, linear or angular velocity, acceleration, or orientation within a certain reference frame, as well as a combination thereof, can be of interest. In general, motion capturing (or MoCap) systems can be divided into optical systems and systems based on inertial sensors, but less common MoCap systems based on mechanical, magnetic, or stretch sensors also exist, with each of the various methods themselves coming with a myriad of different techniques. After a short introduction of optical and inertial systems, along with a brief discussion of the advantages and disadvantages of both, we will focus on inertial MoCap systems. More specifically, we will focus on orientation signals measured by inertial MoCap systems and propose a technique to overcome a certain limitation of these.

**Figure 1:**
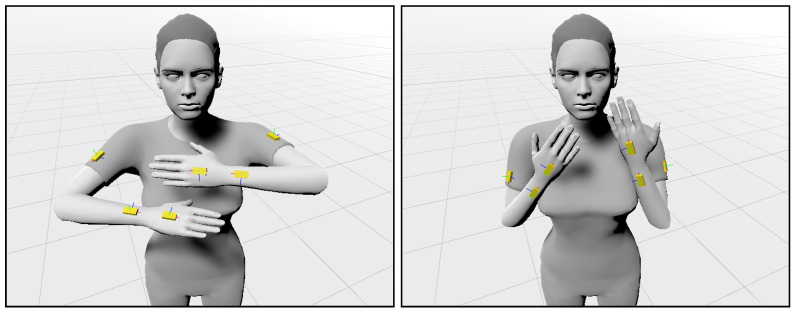
Motion capturing animation from limb-mounted IMU sensors. Horizonal (**left**), Vertical (**right**).

Traditional motion capturing techniques are based on optical systems that track the position of fiducial markers, each carefully placed on the torso and limbs of the person to be tracked, from different viewing angles, by using an arrangement of multiple cameras. While these optical systems are the most accurate, they also tend to be expensive, require a meticulous setup of the cameras, along with a calibration procedure, are susceptible to occlusion, and constrain the motion to be captured to a certain working volume that typically needs to be indoor. Within the last decade, motion can also be captured from a single depth camera [1], or more recently, using deep learning approaches, even from a single RGB camera, e.g., as in [2,3]. An alternative to optical systems are motion capturing systems based on inertial measurement units (IMUs). State-of-the-art IMUs are able to calculate their orientation in space by fusing sensor signals from their accelerometer, gyroscope, and magnetometer. By equipping a person’s limbs and movable body parts with these devices, the person’s posture and movement can be reconstructed. An example is depicted in Figure 1. In general, IMU-based motion capturing systems are less accurate than optical ones, but they are relatively cheap, occlusion-free, and not limited to a certain working volume or an indoor environment. Early work in the research field of wearable computing has investigated dense networks of inertial sensors for interaction [4] and making computers aware of their context [5]. More recent works [6] show that with only 6 IMUs a full body pose can be reconstructed, avoiding the need of wearing and setting up a separate IMU for every limb. There also exist techniques to identify the limb an IMU is attached to by comparing its orientation estimates to those coming from an optical motion capturing system, allowing the combined system to benefit from the complementary advantages of both [7]. To avoid complex and distracting wiring, IMU-based motion capturing systems often consist of several stand-alone sensor units with wireless communication capacities that only need to be attached at the correct body positions. Beyond a lower accuracy and limited position tracking capabilities, their main drawback in this case is a limited battery capacity.

A convenient way to increase the system lifetime, while keeping a reasonable sampling rate of about 50 Hz or higher, is to reduce the amount of data to be transmitted. While the position, velocity, and acceleration of an IMU can unambiguously and efficiently be expressed as a vector in cartesian coordinates, for orientation estimates, there exist various alternatives, such as Euler or Tait-Bryan angles, rotation matrices, axis-angle representation using Rodrigues’ rotation formula, or unit quaternions. In most cases, they are expressed as unit quaternions, which, in contrast to Euler or Tait-Bryan angles, do not suffer from gimbal lock, are more compact than a rotation matrix, and are mathematically more elegant than axis-angle representations. Still, since sensor sampling and communication has to be performed at appropriate rates in order to provide for high resolution motion trajectories, a substantial amount of data needs to be transmitted. At the same time, high sampling and communication rates of wireless low-power networks, e.g., Bluetooth Low Energy (BLE), nevertheless impose a drastic increase in total device energy consumption [8,9]. Since human motion is rarely continuously performed at high rates of change, a considerable amount of energy is consumed by transmitting sensor samples with little to no additional information, if, e.g., the human is standing still. As a consequence, this paper investigates a possible data reduction and, thus, energy saving approach for wearable orientation sensors by using Piecewise Linear Approximation (PLA) techniques. Piecewise linear approximation is a technique that approximates time series signals with linear segments that are guaranteed to be bound by a user-defined upper segment error. In the context of sensor signals in general, PLA techniques have successfully been applied to extract and represent the characteristic signal information recorded by Inertial Measurement Units for the purpose of activity and gesture recognition. Prominent solutions include recognition techniques by dense motif discovery, as introduced in [10], or continuous string and sequence matching algorithms, as introduced in [11,12], respectively.

While PLA techniques, as mentioned above, have successfully been applied to one-dimensional or multi-dimensional data, such as acceleration or angular velocity, to date, and to the best of our knowledge, they have not been applied to orientation data or to quaternion-based signals in particular in the literature. Hence, as a major contribution, the paper at hand investigates the application of PLA algorithms to orientation sensor signals, in order to reduce the amount of data and, thus, energy consumption of wearable sensor nodes in motion capturing scenarios with IMU sensors. We will show that additional requirements for a possible PLA are necessary when dealing with unit quaternions, such as producing segment points that are a subset of the original sensor samples. The rationale behind this will be explained in Section 4 in more detail. Furthermore, a successful interpolation of linear segments on the receiver side necessitates a piecewise linear approximation of connected segments. As a result, PLA techniques that produce connected linear segments are of importance.

While fast and scalable PLA algorithms exist that can be implemented on wireless sensor nodes or even directly on their sensor sub-systems, these do not adhere to the aforementioned requirements. In turn, other existing PLA algorithms that do adhere to these requirements do not provide for an efficient processing of the sensor signals. This gap in the state-of-the-art is evaluated by the paper at hand, and a new online PLA algorithm combining efficient and scalable performance with the ability to approximate quaternion-based orientation sensor signals is proposed and evaluated.

The main contributions of this paper can be summarized as follows:

- A comparison and summary of state-of-the-art PLA methods with emphasis on efficient approximation of quaternion-based orientation sensor signals.
- An evaluation and discussion of the key requirements for applying PLA algorithms to orientation data expressed as unit quaternions.
- A novel PLA algorithm that is scalable, efficient in execution time and memory utilization, and that is suitable to approximate orientation data in the form of unit quaternions.

The remainder of the paper is structured as follows. In the following section, related work is reviewed and discussed in the context of piecewise linear approximation of orientation sensor signals. In Section 3, orientation sensor signals and their common representation by quaternions, as well as their implied requirements on possible PLA techniques, are discussed. Existing PLA techniques will be applied to a selected motion capturing dataset, and their limitations will be evaluated in that section, as well. Section 5 introduces our novel PLA technique, referred to as *fastSW*, which combines time and memory efficient processing capabilities, as well as required segment point selection, for a successful approximation of quaterion-based orientation sensor signals for motion capturing applications. In Section 6, fastSW is experimentally evaluated and formally analyzed w.r.t. its approximation quality and its execution time on resource constrained architectures in comparison to existing PLA methods. Finally, the acquired results are summed up, and conclusions are drawn, in Section 7.

## 2. Related Work

In past decades, several piecewise linear approximation algorithms, sometimes referred to as *segmentation algorithms*, have been introduced in the literature. Especially in sensor-based applications, PLA is often used to reduce the amount of data that has to be stored, transmitted, or further processed, without loosing general trajectory information. A reduction in data size can lead to a reduced energy consumption, e.g., of wireless sensor nodes [8,13]. In order to reduce the total device energy consumption, the reduction due to decreased data size for transmission or storage has to outweigh the increase of energy consumption due to added workload computing the PLA itself [9].

In [14], Keogh et al. introduced the Sliding Window and Bottom Up (SWAB) algorithm, combining the online feature of Sliding Window (SW) and the buffer-based knowledge of future sensor samples from Bottom Up (BU), both well known PLA techniques themselves. Improvements to SWAB have been introduced in [12] (mSWAB) and [15] (emSWAB). However, emSWAB has an average execution time that is magnitudes higher than other PLA algorithms, e.g., SW, which is further scaling with the anticipated segment length. Even SW itself has an O(n) computational and memory complexity of adding a new sample to the PLA w.r.t. the segment length. In [16], a new PLA algorithm, i.e., Connected Piecewise Linear Regression (CPLR), has been introduced, that can be executed in O(1) time for processing a new sensor sample and O(1) memory consumption, making it suitable to be executed on architectures with harsh memory and timing constraints. Another PLA algorithm that can be executed in O(1) time per processed sample and O(1) memory consumption is the Swing Filter (SF), introduced by Elmeleegy et al. in [17]. Both PLA algorithms, CPLR and Swing Filter, make use of the same updating technique for the determination of the best fitting slope of PLA segments; however, the Swing Filter finally alters this choice by an additional restriction by introducing a maximum deviation of single samples as termination condition of a segment. The CPLR algorithm instead, further exploits the updating mechanism and calculates the Sum of Squared Residuals (SSR) error of the segment in O(1) time for each new sample, for which a user defined threshold value provides the termination condition of segments. As a result, both algorithms differ in their error metric. Although within a maximum segment error bound, CPLR and Swing Filter extrapolate segment points from a regression line. As a result, segment points generally do not represent, except by chance, samples of the original signal.

Other PLA algorithms that can be found in the literature include PLAMLiS, introduced by Liu et al. in [18], and its optimization, introduced by Pham et al. in [19]. The computational complexity of processing a single sensor sample, which is decisive for an online approximation of the signals on resource constrained architectures, is not detailed in both works. Their computational complexities of approximating an entire series of *m* sensor samples is given with $O(m^{2}\;log\;m)$ and $O(m^{2})$, respectively. As a consequence, the processing of a single sensor sample is not constant but depends on the length of a buffer, which further constraints their compression abilities and, therefore, possible energy savings.

Lemire introduced a fast PLA algorithm in [20], which, on the other hand, comes at a significant increase in memory consumption. Since an effective energy saving necessitates a computation on a wireless sensor node microcontroller or even on the sensor sub-system itself, the harshly constrained availability of memory prevents a successful application in this domain.

In [21], a segmentation framework based on polynomial least-squares approximation has been introduced by Fuchs et al., referred to as SwiftSeg. Their framework includes first order polynomials, i.e., linear segments, which can produce PLA signals, as well. The first order variant is closely related to the regression principle of CPLR, with the difference that it resembles a linear regression with an intercept term, which produces disconnected segments. CPLR instead, is based on linear regression without an intercept term, which allows it to produce connected segments.

In [22], Luo et al. introduced a PLA algorithm with constant update time, but which is buffer-based. While the processing of a single sample is constant, the memory complexity is linear in the segment length, as their algorithm incorporates a buffer. As a buffer introduces a limit on the segment length, the data compression ability is constrained, and potential energy savings cannot fully be utilized. Furthermore, the introduced PLA algorithm by Lui et al. produces a mixture of connected and disconnected segments.

In the paper at hand, existing PLA algorithms are compared w.r.t. their ability of approximating quaternion-based orientation sensor signals for energy efficient motion capturing applications online on resource constrained architectures, which can be found on wireless sensor nodes and their equipped sensor sub-systems. Furthermore, a novel PLA algorithm is proposed, i.e., fastSW, that unifies the advantages of state-of-the-art PLA methods, w.r.t. approximation quality, choice of segment points, execution time, and memory consumption.

A comparison of state-of-the-art PLA algorithms from the literature, including fastSW, is summarized in Table 1. The first column specifies the PLA methods and its origins, followed by the second column (OL), which denotes the online feature. This is necessary for an application in motion capturing scenarios with wearable sensors, approximating the sensor data on the microcontroller of the wearable or its sensor sub-system itself, online at run time. The third column (CS) denotes if the PLA algorithm produces connected segments, which is necessary for a seamless interpolation of the approximated signals at the receiver side. The fourth column denotes the preservation of sensor samples (POS), i.e., if the PLA segment points are a subset of the original sensor samples. This is a crucial point for quaternion-based signals, which will be thoroughly explained in the next section. The fifth column (BB) lists, whether the PLA algorithms are buffer-based. This constrains in most cases the segment length and, thus, the compression ratio that can be achieved. The segment or sample error metric (EM) listed in the sixth column, specifies the metric that is used for the user-specified error bound of the approximation. The seventh and eighth column list the time and memory complexities of processing a single sensor sample w.r.t. the lengths of a segment *n*, respectively. These are crucial to be small, i.e., ideally O(1), in order to provide for an online application without constraining the compression ratio due to limited computational resources. Correspondingly, the ninth and tenth column, specify the time and memory complexity of processing an entire sequence of samples, w.r.t. the length of that sequence *m*. Here, the buffer size, if applicable, is assumed to be of length *m*, as well, as this allows for maximum compression ratio.

## 3. Quaternion-Based Orientation Sensor Signals

In many fields of computer science, orientations are often represented as unit quaternions. Unit quaternions do have several benefits over other mathematical representations of orientation or rotation. In contrast to Euler angles, they do not suffer from gimbal locks, and they are more compact in memory space as compared to rotation matrices. Moreover, they can be processed more efficiently than rotation matrices. Chaining two rotations, i.e., multiplying two rotation matrices requires 45 operations (27 multiplications and 18 additions/subtractions), while, for unit quaternions, only 28 operations (16 multiplications and 12 additions/subtractions) are required. Furthermore, due to accumulating rounding errors after several processing steps, for instance, in forward or inverse kinematics, the resulting quaternion or matrix may not represent a valid orientation anymore. A quaternion can easily be converted back to a valid orientation representation by normalizing it, while converting a matrix back to a proper orthogonal rotation matrix is much harder to achieve. Another desirable property of unit quaternions is that they provide with spherical linear interpolation (SLERP) an easy way to smoothly transition or interpolate from one orientation to another along their geodesic, i.e., the shortest arc between two points on a hypersphere.

Typical applications where unit quaternions are used range from computer animation and motion capturing over robotics up to space craft and airplane or satellite navigation. A promising application for piecewise linear approximation of quaternion signals hereby is the field of computer graphics. While man-made animations typically consist of a couple of keyframes that are interpolated (often using animation curves) to achieve a fluid animation, motion capturing (MoCap) data consists of an array of individual orientation samples of all joints captured at the given sampling rate. A motion capturing file, thus, consumes much more memory than an animation file, while not necessarily providing more detailed motion features. For instance, not all limbs are moving at the same time, and a limb movement may also sufficiently be expressed as a (spherically) linear movement between various limb angles. Being able to reduce the size of a MoCap file to only contain the necessary keyframes that mark a change in a limb movement and to completely remove all static joints, thus, has the potential to significantly reduce the memory requirements of such a file. For IMU-based MoCap systems, the data reduction, furthermore, may directly happen on the sensing devices and can reduce both the required data traffic and, thus, the device power consumption. This also extends to telemedicine and rehabilitation scenarios, e.g., gait evaluation [23,24], that is based on capturing the movement of limbs via wearable IMU sensors. In the next section, state-of-the-art PLA algorithms and their limitations for quaternion-based orientation sensor signals will be discussed.

## 4. Piecewise Linear Approximation of Quaternion-Based Orientation Sensor Signals

In order to evaluate the approximation quality of existing PLA algorithms w.r.t. quaternion-based orientation sensor signals, the public TNT15 dataset [25] works as a case study for the paper at hand (see Section 6).

Since approximating sensor data is prone to information loss, one of the quality indicators of an approximation technique is the approximation error. This error is adjustable by a user defined threshold in terms of a maximum SSR error per segment (SW, CPLR, SWAB, mSWAB) or a maximum absolute residual error per sample (SF). Another quality indicator of PLA algorithms is the ratio to which sensor data can be compressed. A higher compression leads to fewer segment points that have to be transmitted wirelessly or stored in the flash memory of wearable sensor nodes. In the paper at hand, the inverse of the compression ratio is used as the second quality indicator, which is the ratio between the size of the resulting PLA signal $\tilde{S}$ and the original signal *S*. As a result, a small *inverse compression ratio* (ICR) is desirable when approximating sensor signals, in order to reduce the amount of data to be transmitted, thus saving energy of the wearable device. The ICR is calculated by:

(1) $$ICR=\frac{\tilde{m}}{m}\;,$$

with $\tilde{m}$ denoting the length of the PLA signal $\tilde{S}$, and *m* denoting the length of the original signal *S*. Although not linearly or monotonically dependent, the general trend is a higher approximation error at lower ICRs. As an indicator of overall approximation quality, both, approximation error and ICR, have to be traded-off.

While, in [16], the approximation error for several inertial sensor signals has been evaluated as the sum of squared residuals error of the approximated dataset, the evaluation in the paper at hand is focused on orientation sensors. As a result, the approximation error can naturally be expressed in terms of an angle $\Delta (q_{0},q_{1})$ between two quaternions $q_{0}$ and $q_{1}$, which correspond to the original sensor sample and its approximation. The angular deviation of two quaternions $q_{0}$ and $q_{1}$ is calculated by:

(2) $$\Delta \left(q_{0},q_{1}\right)=2\arccos\left|\langle q_{0},q_{1}\rangle \right|\;,$$

with $\left|\langle q_{0},q_{1}\rangle \right|$ denoting the absolute value of the dot product between $q_{0}$ and $q_{1}$ considered as 4 component vectors. Hence, the approximation error is defined as the sum of absolute angular deviations w.r.t. the shortest rotation between individual samples of the original sensor signal and its PLA interpolated at the corresponding timestamps of the original signal. Since the lengths of the evaluated dataset scales the sum of angle deviations, the result is further normalized by the lengths of the original signal, hence resulting in an *Average Angular Deviation* (AAD), which is calculated as follows:

(3) $$AAD=\frac{1}{\begin{array}{c} m \end{array}}\sum_{i=0}^{m-1}\Delta \left(S\left[i\right],{\tilde{S}}^{\prime }\left[i\right]\right)\;,$$

with ${\tilde{S}}^{\prime }$ being the PLA signal $\tilde{S}$ interpolated at the corresponding timestamps of the original signal *S*, $\begin{array}{c} m \end{array}$ being the lengths of both signals, and Δ(x,y) being the angular deviation between two quaternions. In the paper at hand, the interpolated PLA signal ${\tilde{S}}^{\prime }$ is based on SLERP, a standard method to interpolate between two unit quaternions. It has the benefit of the fact that interpolation happens along the geodesic (or shortest arc) of the quaternion hypersphere, and, at the same time, the angular velocity of the resulting rotation is retained.

In order to express a 3D orientation, a quaternion needs to be of length one; hence, it needs to be a unit quaternion. Quaternions that do not have unit length need to be normalized before further processing; otherwise, for instance, a vector being rotated by a quaternion would also be scaled with the scale error being propagated by the square of the quaternion norm. Furthermore, spherical linear interpolation would not yield correct results when quaternions do not have unit length. While the orientation sensor signals acquired from IMUs are composed of unit quaternions, the application of PLA algorithms to these signals may produce segment points that deviate from unit quaternions. This is generally the case with PLA algorithms based on linear regression, which do not preserve original signal values in their resulting PLA (cf. column POS in Table 1) but extrapolate segment points from regression lines. Examples of linear regression-based PLA algorithms are CPLR and SF. The extrapolation of segment points from a regression line scales the signal unequally among its axes, which results in an unwanted rotation when normalizing the quaternion. This results in a higher angular deviation of segment points compared to their original signal values at higher compression ratios when approximated with regression-based PLA algorithms, such as CPLR and SF.

**Figure 2:**
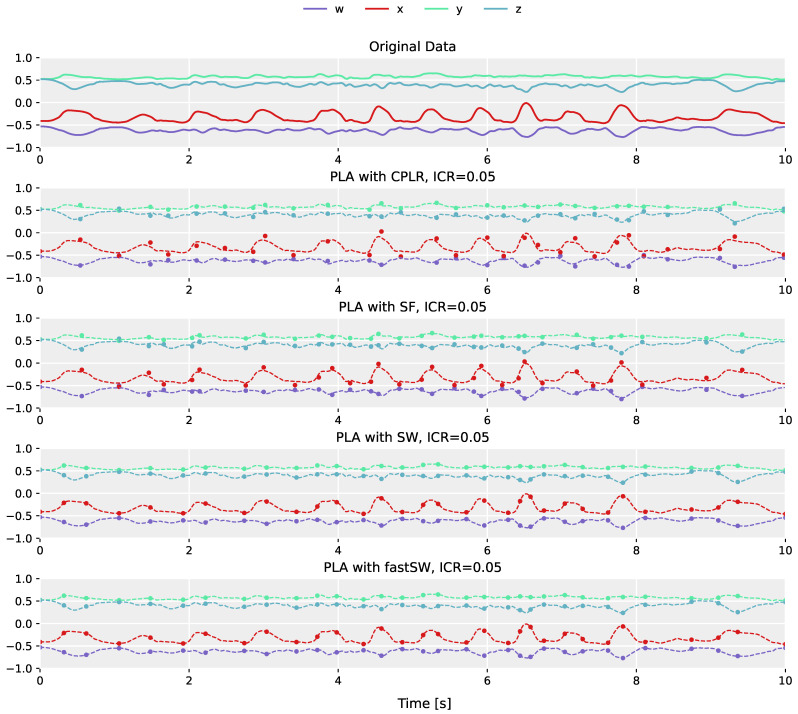
Example of the different PLA method outputs at a compression ratio of approximately 5% the size of the original data. Shown is the quaternion data from the left shank of the fast-paced *running on spot* activity of user *MR* from the TNT15 dataset, with the original data at the top, and the produced segment points overlaid over the original data by the algorithms (from top to bottom): CPLR, SF, SW, and fastSW (our proposed method). CPLR and SF produce segment points that are not a subset of the original data, while SW and fastSW produce segment points that lie on the original data.

The differences in segment point construction between regression-based PLA algorithms (e.g., CPLR and SF), and PLA algorithms that produce segment points that are a subset of the original sensor data (e.g., SW), is demonstrated in Figure 2. The plot in the top row shows the original orientation data obtained from the left shank of the fast-paced *running on spot* activity of user *MR* from the TNT15 dataset. The following plots below (from top to bottom) depict the corresponding PLAs produced by CPLR, SF, SW, and our proposed method (fastSW) at a similar inverse compression ratio of approximately 5%. For the sake of comparison, PLAs are depicted as point markers overlaid over the original sensor data represented by dashed lines. The segment points of CPLR and SF do not lie on the original data, except by chance. When approximated by SW (or fastSW), segment points are guaranteed to lie on the original data, since SW selects a subset of the original signal values as its segmentation points. This will be explained in more detail in Section 5.

Due to the fact that SW preserves original sensor samples in its resulting PLA and, thus, retains unity of produced segment points, it can be considered as an appropriate PLA algorithm for orientation sensor signals represented by quaternions. Nevertheless, although being one of the most efficient state-of-the-art PLA algorithms in terms of execution time and memory consumption (cf. columns TCn, MCn, TCm, and MCm in Table 1) that preserve original signal values within the PLA (cf. column POS in Table 1), the major drawback of SW is its linear execution time and memory complexity per sensor sample w.r.t. the lengths of the produced segments and, thus, its effective compression ratio. This increases the processor utilization for higher compression ratios, which might outweigh the data rate reduction in terms of energy consumption, depending on the deployed architecture.

In the paper at hand, we introduce a new PLA algorithm which is based on SW but exploits the SSR updating technique from CPLR, which ultimately leads to mathematically equal PLA results to those acquired by SW, but with a O(1) computational and memory complexity for processing a new sample, such as CPLR and SF. This novel PLA algorithm is referred to as *fastSW* and is introduced in the following section.

## 5. Efficient Piecewise Linear Approximation with fastSW

The general procedure of our proposed algorithm is based on the original SW algorithm: for each new (*n*-th) sensor sample, a linear function (temporary segment) β·t is created based on the current sensor sample $s_{n}$ and the last segmentation point ${\tilde{s}}_{i-1}$, with *t* denoting the length in time of the temporary segment, i.e., $t=\tau \left(s_{n}\right)\;-\;\tau \left({\tilde{s}}_{i-1}\right)$, with τ denoting the timestamp part of a sample or segmentation point, respectively. To this end, the slope vector entry $\beta_{d}$ for each dimension d=1,⋯,D of the *D*-dimensional segment is calculated by:

(4) $$\beta_{d}=\frac{\upsilon \left(s_{n},d\right)-\upsilon \left({\tilde{s}}_{i-1},d\right)}{t}\;,$$

with υ(s,d) denoting the amplitude value of a sample *s* in dimension *d*. Note that *n* is the index of the newest sample within the currently developing segment, which is reset to one with each newly created segment point.

The SSR error is calculated between the temporary segment with slope vector β, and the original signal samples between ${\tilde{s}}_{i-1}$ and $s_{n}$. The segment SSR up to the *n*-th sample of the current segment $\left({\tilde{s}}_{i-1},s_{n}\right)$ is mathematically defined by:

(5) $$SSR_{n}=\sum_{j=1}^{n}\sum_{d=1}^{D}{\left(y_{dj}-\beta_{d}\cdot t_{j}\right)}^{2}\;,$$

with $y_{dj}$ representing the amplitude of the *j*-th sensor sample of dimension *d* within the segment coordinate system, i.e., $y_{dj}=\upsilon \left(s_{j},d\right)-\upsilon \left({\tilde{s}}_{i-1},d\right)$, and $t_{j}$ representing the timestamp of the *j*-th sensor sample within the segment coordinate system, i.e., $t_{j}=\tau \left(s_{j}\right)-\tau \left({\tilde{s}}_{i-1}\right)$.

The original SW algorithm buffers all original samples spanning the temporary segment, i.e., $s_{1},\cdots ,s_{n}$, and iterates over these in order to calculate the segment SSR error by Equation (5). This iteration causes a linear Worst-Case Execution Time (WCET) for processing a single new sensor sample w.r.t. the temporary segment lengths.

Similar to CPLR [16], fastSW calculates the SSR error of the temporary segment based on updated values, in constant time. To this end, binomial expansion is applied to the accumulated term in Equation (5), and the two commutative sums are swapped, which results in:

(6) $$SSR_{n}=\sum_{d=1}^{D}\left(\sum_{j=1}^{n}\left(y_{dj}^{2}\right)-2\beta_{d}\sum_{j=1}^{n}\left(t_{j}\cdot y_{dj}\right)+\beta_{d}^{2}\sum_{j=1}^{n}\left(t_{j}^{2}\right)\right)\;.$$

Each sum over all *n* samples of a segment can now be updated with each new sample in a constant time. Furthermore, the accumulation over the signal dimensions is independent of the segments length. However, depending on the size of the segment and the amplitude range of the sensor signal, the updated sums can span a rather large range of values, which can cause numerical issues in fixed or floating-point implementations. As a solution, corresponding mean values are stored and updated instead, which substitute the sums in Equation (6), together with a multiplication with *n*, that is:

(7) $${\bar{y^{2}}}_{dn}\cdot n=\sum_{j=1}^{n}\left(y_{dj}^{2}\right)\;,$$

(8) $${\bar{ty}}_{dn}\cdot n=\sum_{j=1}^{n}\left(t_{j}\cdot y_{dj}\right)\;,\;\mathrm{and}$$

(9) $${\bar{t^{2}}}_{n}\cdot n=\sum_{j=1}^{n}t_{j}^{2}\;.$$

The means ${\bar{y^{2}}}_{dn}$, ${\bar{ty}}_{dn}$, and ${\bar{t^{2}}}_{n}$, are updated by the timestamp $t_{n}$ and signal amplitude $y_{dn}$ of the new sensor sample $s_{n}$ within the coordinate system of the currently developing segment (originating in ${\tilde{s}}_{i-1}$), by:

(10) $${\bar{y^{2}}}_{dn}={\bar{y^{2}}}_{dn-1}+\frac{{\bar{y^{2}}}_{dn-1}-y_{dn}^{2}}{n}\;,$$

(11) $${\bar{ty}}_{dn}={\bar{ty}}_{dn-1}+\frac{{\bar{ty}}_{dn-1}-t_{n}\cdot y_{dn}}{n}\;,\;\mathrm{and}$$

(12) $${\bar{t^{2}}}_{n}={\bar{t^{2}}}_{n-1}+\frac{{\bar{t^{2}}}_{n-1}-t_{n}^{2}}{n}\;.$$

By assuming a constant dimensionality of the signals to be approximated (which is the case with state-of-the-art inertial and orientation sensor data), the segment error $SSR_{n}$ up to the *n*-th sample can then be re-calculated from the updated means for each newly included sensor sample $s_{n}$ in a constant number of steps, by:

(13) $$SSR_{n}=\sum_{d=1}^{D}\left({\bar{y}}_{dn}^{2}-2\beta_{d}{\bar{ty}}_{dn}+\beta_{d}^{2}{\bar{t^{2}}}_{n}\right)\cdot n\;.$$

Note that the calculation of $SSR_{n}$ differs from CPLR (Equation (22) in [16]), as $\beta_{d}^{2}$ cannot be resolved here since, for fastSW, $\beta_{d}$ is not representing the slope of a regression line of the original samples but the slope from the last segment point and the current sample, as shown in Equation (4).

With the aforementioned equations, the fastSW algorithm can now be introduced, the execution time of which is independent of the temporary segment length. The pseudo-code of fastSW is given in Algorithm 1.
**Algorithm 1** fastSW.1:**procedure**PROCESS_SAMPLE(sample *s*, segment array $\tilde{S}\left[\right]$, index *i*)2:      n=n+13:      $SSR_{n}=0$4:      $t_{n}=timestamp\left(s\right)-timestamp\left(\tilde{S}\left[i-1\right]\right)$5:      **for** *d* in (1,⋯,D) **do**6:            $y_{n}\left[d\right]=value\left(s,d\right)-value\left(\tilde{S}\left[i-1\right],d\right)$7:            $\beta \left[d\right]=y_{n}\left[d\right]/t_{n}\left[d\right]$8:            $SSR_{n}=SSR_{n}+\left({\bar{y^{2}}}_{n-1}\left[d\right]-2\beta \left[d\right]\cdot {\bar{ty}}_{n-1}\left[d\right]+\beta {\left[d\right]}^{2}\cdot {\bar{t^{2}}}_{n-1}\right)\cdot \left(n-1\right)$9:      **if** $SSR_{n}<=TH$ **then**10:           ${\bar{t^{2}}}_{n-1}={\bar{t^{2}}}_{n-1}+\left(\left(t_{n}\cdot t_{n}\right)-{\bar{t^{2}}}_{n-1}\right)/n$11:          **for** *d* in (1,⋯,D) **do**12:             ${\bar{ty}}_{n-1}\left[d\right]={\bar{ty}}_{n-1}\left[d\right]+\left(\left(t_{n}\cdot y_{n}\left[d\right]\right)-{\bar{ty}}_{n-1}\left[d\right]\right)/n$13:             ${\bar{y^{2}}}_{n-1}\left[d\right]={\bar{y^{2}}}_{n-1}\left[d\right]+\left(\left(y_{n}\left[d\right]\cdot y_{n}\left[d\right]\right)-{\bar{y^{2}}}_{n-1}\left[d\right]\right)/n$14:          $s_{n-1}=s$15:          return 016:      $\tilde{S}\left[i\right]=s_{n-1}$17:      $s_{n-1}=s$18:      n=119:      $t_{n}=timestamp\left(s\right)-timestamp\left(\tilde{S}\left[i\right]\right)$20:      ${\bar{t^{2}}}_{n-1}=t_{n}\cdot t_{n}$21:      **for** *d* in (1,⋯,D) **do**22:            $y_{n}\left[d\right]=value\left(s,d\right)-value\left(\tilde{S}\left[i\right],d\right)$23:            ${\bar{ty}}_{n-1}\left[d\right]=t_{n}\cdot y_{n}\left[d\right]$24:            ${\bar{y^{2}}}_{n-1}\left[d\right]=y_{n}\left[d\right]\cdot y_{n}\left[d\right]$25:      return 1

The very first sensor sample needs to be stored in $\tilde{S}\left[1\right]$ as the initial segment point. For each following sensor sample $s_{j}$, function PROCESS_SAMPLE is executed with $s_{j}$ as the first parameter, the segment array $\tilde{S}\left[\right]$, which needs to be at least of size two, and the index *i*, at which the next segment point will be stored within $\tilde{S}\left[\right]$.

The variables *n*, TH, and *D*, as well as the vectors ${\bar{ty}}_{n-1}\left[\right]$ and ${\bar{y^{2}}}_{n-1}\left[\right]$, both of size *D*, and the previous sensor sample $s_{n-1}$ need to be stored globally. All other variables are temporary. The variables TH and *D* for the threshold and dimensionality of the sensor signal, respectively, need to be set at initialization. Variable *n* and vectors ${\bar{ty}}_{n-1}\left[\right]$ and ${\bar{y^{2}}}_{n-1}\left[\right]$ are initialized with zero.

At the beginning of Algorithm 1 in line 2, the current segment length *n* is incremented due to the new sensor sample, which is temporarily assumed to be covered by the currently developing segment. In line 3, the segment error is reset to zero. The timestamp $t_{n}$ of *s* within the coordinate system of the current segment is calculated in line 4, based on the last segment point $\tilde{S}\left[i-1\right]$ and *s*. Note that function timestamp(s) returns the timestamp part of a sensor sample or a segment point *s*, respectively. The amplitudes of *s* in the coordinate system of the current segment are calculated for each of the signal dimensions (line 5) and stored in the vector $y_{n}\left[\right]$, in line 6. Note that function value(s,d) returns the amplitude in dimension *d* of a sensor sample or a segment point *s*, respectively. The slope of the current segment is calculated for each signal dimension and stored in vector β[] in line 7, and the SSR of each signal dimension between the current segment and the original sensor data covered by that segment is calculated and accumulated into the total segment error $SSR_{n}$, in line 8.

If the SSR error of the current segment is smaller than or equal to the user-defined threshold TH (line 9), the new sample *s* is manifested within the current segment, by updating the running variable ${\bar{t^{2}}}_{n-1}$ and the vectors of running variables ${\bar{y^{2}}}_{n-1}\left[\right]$ and ${\bar{ty}}_{n-1}\left[\right]$ in lines 10, 12, and 13, respectively. The new sample is stored in $s_{n-1}$ in line 14, in case a new segment needs to be created from there, in the next invocation. Finally, the function returns with zero in line 15, indicating, that no new segment has been created.

In case $SSR_{n}$ is greater greater than TH (line 9), the sensor sample added in the previous function invocation, i.e., $s_{n-1}$, will be set as new segment point at position *i* in array $\tilde{S}\left[\right]$, in line 16. Since the new sensor sample $s_{n}$ is already the first sample to be covered by the newly started segment and also its current end point, it is stored in $s_{n-1}$ for the next function invocation, in line 17, and the number of samples *n* is reset to one in line 18. The timestamp of *s* within the coordinate system of the newly started segment is calculated in line 19, with which the running variable $t_{n-1}$ is re-initialized for the newly started segment in line 20.

Similarly, the amplitudes of *s* in the coordinate system of the newly started segment are calculated for each dimension in line 22, with which also the vectors of running variables ${\bar{y^{2}}}_{n-1}\left[\right]$ and ${\bar{ty}}_{n-1}\left[\right]$ are re-initialized in lines 23 and 24, respectively. The function returns with 1 in line 25, indicating that a new segment point has been created. The new segment point is stored in $\tilde{S}\left[i\right]$. After function PROCESS_SAMPLE returns, the new segment point should either be moved to position i−1 in $\tilde{S}\left[\right]$, in case the latter is of size two, or *i* should be incremented with the next invocation of PROCESS_SAMPLE due to a new sensor sample, in case $\tilde{S}\left[\right]$ is supposed to store the entire or a batch of created segment points. Ultimately, management of segment points is dependent on the anticipated application and should be decided by the system designer.

Similar to other state-of-the-art segmentation algorithms, the time and memory complexity of fastSW is linear w.r.t. the dimensionality of the signal (lines 5 and 11 in Algorithm 1). The dimensionality, however, can commonly be assumed to be constant. More importantly, the time and memory complexity of fastSW w.r.t. the segment length and, thus, the compression ratio is constant. SW, in contrast to fastSW, shows a linear time and memory complexity w.r.t. the segment lengths. Both will be shown in the following section.

## 6. Experimental Evaluation

In order to assess the performance of our proposed method, we selected the methods CPLR, SF, and SW to be compared against fastSW. All methods have in common that they provide PLAs with connected segments and can be considered the most efficient state-of-the-art PLA algorithms in terms of computational and memory complexity (cf. columns CS, TCn, and MCn in Table 1).

For the application on quaternions, all PLA algorithms have been implemented to process 4D sensor data. Furthermore, the maximum segment length is set to 1000 samples. Although CPLR, SF, and fastSW do not require a limited segment length (cf. line 9 in Algorithm 1), it is implemented to ensure comparable results to SW. As a consequence, the minimum achievable ICR in our experiments is limited to 0.001, or 0.1%.

Each PLA algorithm can be controlled by a user-specified threshold value on its internal error metric, but the threshold merely constitutes a control parameter, rather than a quality indicator. Due to the differences in their concept, different algorithms may produce PLAs with different approximation errors and compression ratios at the same threshold, while they may reach the same compression ratio and approximation error at different thresholds. Hence, PLA results cannot be compared based on equal threshold values, but approximation errors have to be compared at similar compression ratios, which will be considered in our evaluation on real data.

The experimental evaluation further is divided into three parts. In a first experiment, the computational complexities of the selected PLA algorithms on a real world example are investigated. Then, a WCET analysis on a representative architecture (ARM Cortex-M4) is performed, and, in a third and final experiment, the approximation quality of all methods is assessed and compared.

The first and third experiment are evaluated on the publicly available TNT15 dataset [25]. It comprises 7 activities performed by 4 different actors, summing up to a total of 28 different recordings. The activities include walking, running on the spot, rotating arms, jumping and skiing exercises, dynamic punching, and two activities that are not further specified in the dataset documentation. Each recording, furthermore, was performed with 10 IMUs placed at the shanks, thighs, lower arms, upper arms, neck, and hip, with the sensors providing acceleration and orientation data at a sampling rate of 50 Hz. Overall, the dataset comprises 4040 to 10,180 samples per file, from which the acceleration data is discarded, and only the orientation data in the form of unit quaternions is used. This ensures avoiding distortion in our evaluation results by artifacts from additionally processing acceleration data, for which existing PLA techniques already have proven to work well.

Since it is not guaranteed to reach exactly equal compression ratios with different PLA algorithms on a particular dataset, multiple PLAs need to be produced with varying threshold values for each evaluated algorithm. To this end, each of the 28 recorded files in the dataset has been approximated at 205 different threshold values, starting at zero and logarithmically increasing evenly among 9 magnitudes, ranging from 0.000001 to 1000, in order to acquire an appropriate coverage of ICRs for CPLR, SF, SW, and fastSW, respectively. This applies to both, the first and the third experiment. Details on the experimental design and the acquired results from the three experiments are presented in the following sections.

### 6.1. Execution Time Measurements on an x86_64 Processor

In order to observe the execution time dependency on the segment length in a real world example, we have measured the execution times of the selected PLA algorithms for processing each single sensor sample in the TNT15 dataset. Since, for computational complexity, the absolute timing is not relevant, but the growth in execution time w.r.t. a certain parameter (in this case the average segment length), these measurements have been performed on a standard x86_64 computer architecture. This setup, furthermore, simplifies automated processing of all files within the dataset.

The execution of each invocation for a new sensor sample has been measured by taking timestamps before and after the corresponding function to process a single sensor sample. The implementation is based on the clock_gettime syscall of the Linux kernel in version 5.12.9 with CLOCK_MONOTONIC_RAW as clock source and a resolution of 1 ns. The experiments have been performed on an Intel Core i7-5600U CPU, and the algorithms have been compiled with the GNU Compiler Collection (GCC) C compiler in version 11.1.0 [26].

**Figure 3:**
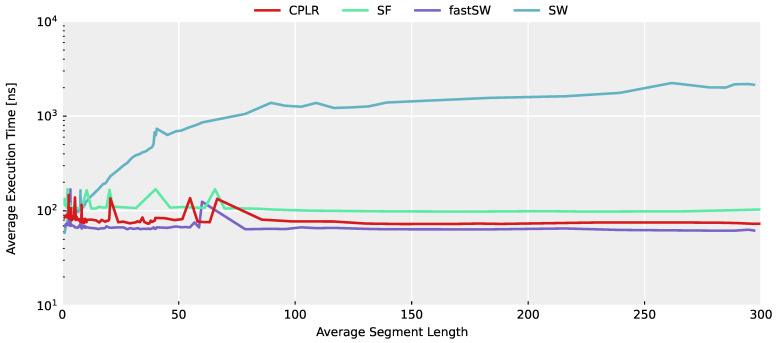
Average execution time per sample plotted against the resulting average segment length of each PLA algorithm applied to the entire TNT15 dataset at a high range of threshold values on an x86_64 architecture. For the sake of comparison, the execution time is plotted in a logarithmic scale. CPLR, SF, and fastSW show a constant execution time, while SW shows a linear average execution time, with respect to the average segment length.

In Figure 3, the average execution time across the entire TNT15 dataset of each algorithm is depicted in relation to the average segment length for each evaluated threshold value. A higher average segment length denotes a higher compression and, thus, a smaller PLA data size. Despite outliers caused by architectural influences (most importantly, cache misses per se, which are further influenced by other scheduled processes), the constant time complexity of CPLR, SF, and fastSW can be seen, as well as the linear execution time growth of SW w.r.t. the segment length. Furthermore, it can be seen that fastSW has the smallest execution time among the PLA algorithms with constant time complexity and outperforms SW at an compression ratio of approximately 2 and upwards, as well.

From this first experiment, the linear execution time growth of SW w.r.t. the compression rate is evident. However, the actual timing results do not reflect expected timing behavior on the target hardware architectures of the anticipated use case, i.e., wireless sensor nodes. As a result, a second evaluation has been designed for evaluating the timing of all algorithms on a representative hardware architecture, i.e., an ARM Cortex-M4 microcontroller.

### 6.2. Worst-Case Execution Time Analysis on an ARM Cortex-M4 Instruction Set Architecture

While the measured execution times on the x86_64 architecture give enough information to assess the computational complexity, their absolute timing is not representative for wearable devices. As a result, the second part of the evaluation is based on a WCET analysis on the ARM Cortex-M4 architecture. Instead of the actual timing, the instruction count for each algorithm is measured. The rationale behind this is three-fold. Firstly, the ARM Cortex-M4 is a representative microcontroller architecture for wearable devices. Secondly, a WCET analysis is not limited to a particular dataset in which the worst case (in terms of execution time) might not be contained. In the particular case, this would include situations in which the sensor is not moved and the produced PLA segments would grow particularly long. While this constitutes a situation in which data can be compressed effectively, algorithms, such as SW, where the execution time is linear dependent on the segment length, would suffer from an increased processor utilization, eventually being limited by the available computational resources. Finally, since instruction set architectures (ISAs), such as the ARM Cortex-M4, can be implemented with different clock frequencies that eventually dictate final execution times, analyses based on instruction counts provide a common base for comparisons of algorithms independent of the architectural implementation of the microcontroller and can, furthermore, be translated to such.

Since the WCET analysis is based on compiled source code for the M4 architecture, each algorithm has been compiled with the Arm Embedded GCC version 11.1.0 of the GNU Arm Embedded Toolchain [26] with the Cortex-M4 chosen as target platform (command line option -mcpu=cortex-m4). Floating-point instructions have been set with Floating-Point Unit (FPU) specific calling conventions (command line option -mfloat-abi=hard), and the optimization level has been set to highest w.r.t. to execution time (command line option -O3). The compiled assembler codes (command line option -S) have been subjected to automated generation of Control Flow Graphs (CFGs) with corresponding instruction counts (ICs) of the basic blocks. The CFGs, due to their simplicity, have been analyzed manually w.r.t. minimal and maximal execution time, as well as their execution time dependency w.r.t. loop counter variables, i.e., segment lengths. The results are summarized in Table 2.

As can be seen in Table 2, the maximal ICs of CPLR, SF, and fastSW are independent of the segment length and, thus, of the compression ratio. Furthermore, CPLR and fastSW show a maximum IC that is approximately half of that of SF. The maximum IC of SW is 161 instructions in general and, for each sample in the buffer (segment length *n*), 35 additional instructions. As an example, for a segment length of 100, SW would execute 3661 instructions for processing a new sample in the Cortex-M4 architecture, while fastSW processes each sensor sample with a maximum of 210 instructions, independent of the segment length. This shows the superiority of the segment error updating mechanism of fastSW compared to SW, while achieving quasi-equal results. The term quasi-equal is used, as there might occur small differences in the segment error calculation due to the numerical precision of floating point operations which are mathematical identical but computed in a different order. As a result, for SW, the segment error threshold might be reached for one sample while fastSW reaches it on the next sample, or vice versa. However, these differences do not significantly influence the approximation quality. The resulting approximations represent operating points which can be reached by both algorithms, due to their constraint of producing segment points that are a subset from the original sensor signal.

### 6.3. Approximation Quality

In this section, the approximation quality of CPLR, SF, SW, and fastSW on the TNT15 dataset is assessed. The dataset has been approximated with the selected PLA algorithms as explained in the beginning of this section, in order to compare resulting approximation errors at similar compression ratios.

The resulting curves from relating approximation errors and compression ratios are compared to each other. By plotting these curves over the ICR, the approximation quality can be assessed by the distance of the resulting curves from the origin on the plot. That is, the nearer the curve to the origin of the coordinate system, the better the approximation quality.

Since the approximation quality not only depends on the particular PLA algorithm itself but also on the time-dependent characteristics of the data, we assessed the long term average, standard deviation, and maximum of the approximation error over the entire TNT15 dataset in order to quantify the approximation quality and its variation.

**Figure 4:**
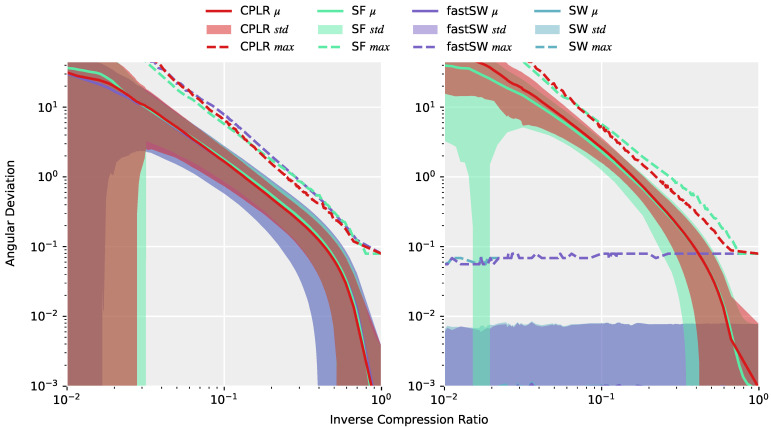
**Left**: Average (solid line), standard deviation (shaded area), and maximum (dashed line) of angular deviations of the approximations of the entire TNT15 dataset with CPLR, SF, SW, and fastSW from the original signal after interpolating the produced segment points using spherical linear interpolation (SLERP). **Right**: Average, standard deviation, and maximum of angular deviations of only the segments points of the same approximations. Values are plotted against the resulting inverse compression ratio (ICR) at a high range of different threshold values. Colors of the shaded regions denoting the standard deviations mix up and may extend over the whole plot at smaller ICRs due to the logarithmic scale of the y-axis. While differences of the methods on the reconstructed signal (**left plot**) are not obvious, the differences of the segment points themselves (**right plot**) are significant. CPLR and SF show increasing angular deviations of their segment points with decreasing ICR. The segment points of SW and fastSW, on the other hand, are not affected by the ICR and show constant angular deviations at a small scale caused by numerical precision.

Figure 4 (left) depicts the angular deviations (or approximation errors) of the approximations of the entire TNT15 dataset after interpolating the produced segment points from CPLR, SF, SW, and fastSW at the corresponding timestamps of the original data using SLERP. All methods exhibit a similar or, in the case of SW and fastSW, nearly the same approximation quality, which means that differences of the methods are not obvious from the reconstructed signal. A completely different picture is drawn on Figure 4 (right), where only the angular deviations of the segment points themselves to the corresponding data points from the original signal are measured. Here, the differences of the methods are significant. CPLR and SF exhibit increasing angular deviations of their segment points with decreasing inverse compression ratio, while SW and fastSW show constant angular deviations at a small scale over the whole range of inverse compression ratios. As expected, and despite numerical differences in regions with a small number of segments (low inverse compression ratio) and numerical precision of the angular deviation per se, both SW and fastSW produce segment points that do not deviate from their original sensor samples. However, PLAs from linear regression-based algorithms, such as CPLR and SF produce extrapolated segment points which deviate from unit quaternions as the compression increases (lower ICRs) and, thus, introduce higher angular deviations between produced segment points and the corresponding original orientation quaternions. In Figure 4 (right), the average angular deviations of segment points produced by CPLR and SF reach up to more than 40° at an ICR of 0.01. In contrast, angular deviations of segment points produced by SW and fastSW are independent of the compression, with their maximum angular deviation staying below 0.1° and their average angular deviation staying below 0.001°.

**Figure 5:**
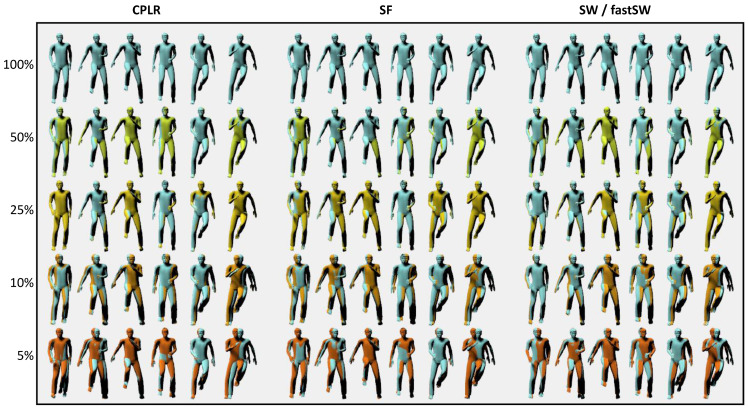
Reconstruction results of the fast-paced *running on spot* activity of user *MR* from the TNT15 dataset. Left: CPLR, Middle: SF, Right: SW/fastSW. Single frames (from left to right) are taken at six different time points every 100 ms. Colors indicate file size w.r.t. the original size and are arranged from top to bottom, with: The original file (light blue), 50% (light green), 25% (yellow), 10% (orange), and 5% (dark orange) of the original file size. Ground truth frames (light blue) are overlaid over all frames to highlight deviations. At 50% and 25%, a good approximation is obtained from all methods. Higher compression ratios yield less accurate reconstructions.

For the sake of demonstration, the reconstruction results of the *running on spot* activity of user *MR* from the TNT15 dataset at six different frames and five different compression ratios are depicted in Figure 5 for CPLR, SF, SW, and fastSW. The frames have been interpolated from the segment points using SLERP. Although the focus of the paper at hand is not particularly on animation or motion reconstruction but rather on motion capturing with orientation sensors, the reconstructed animations in Figure 5 provide a good visual feedback on the trade-off between approximation error and compression ratio.

For animation, a clear benefit of SW and fastSW over other state-of-the-art PLA algorithms cannot be stated, as interpolation between segment points introduces approximation errors that have a higher impact with increasing compression ratio than the segment points. However, in scenarios when orientation sensor data needs to be reduced but accurate supporting points are necessary, e.g., a combination of video and IMU-based motion capturing, SW and fastSW can deliver accurate segment points that do not have to be normalized and, therefore, prevent additional rotation errors. This also translates to other applications.

While SW could offer these properties already, but at a linear computational and memory complexity w.r.t. compression ratio which diminishes possible energy savings due to increased processing, fastSW offers an efficient alternative with a constant computational and memory complexity. Furthermore, since fastSW does not need to buffer sensor data, the segment length and, thus, compression ratio is theoretically not limited, without increasing memory consumption.

## 7. Summary and Conclusions

In the paper at hand, we studied the applicability of state-of-the-art PLA algorithms in motion capturing scenarios where limb orientations are acquired by quaternion-based orientation sensors from wearable devices. Our analysis revealed a gap in the state-of-the-art: There is no computationally efficient PLA algorithm that provides PLA segments that are a subset of the original sensor data. For general IMU applications, deviation from the original data is not a crucial issue. However, unit quaternions are susceptible to that, as demonstrated in our experiments. They are not only required to be normalized again, but even slight deviations from the original data on different axes can also cause significant angular deviations as shown in Section 6.3. We further introduced our novel PLA algorithm fastSW, that is based on the original sliding window algorithm but lends its time and memory efficient updating technique of the segment error from CPLR, allowing it to be performed with a time and memory complexity of O(1) w.r.t. the compression ratio. Hence, fastSW allows for an efficient and effective reduction in data transmissions and, thus, energy consumption, without introducing additional angular deviations to segment points when approximating quaternion-based orientation sensor signals.

## Figures and Tables

**Table 1:** Comparison of state-of-the-art PLA algorithms, with OL denoting online applicability, CS the ability to produce connected segments, POS the preservation of original samples, BB the necessity of a buffer, EM the error metric, TCn the time complexity for processing a single sample w.r.t. the segment length *n*, MCn the memory complexity for processing a single sample w.r.t. segment length *n*, TCm the time complexity for processing an entire sequence w.r.t. to its length *m*, MCm the memory complexity for processing an entire sequence w.r.t. to its length *m*, SSR the sum of squared residuals error of a segment, SAD the sum of absolute deviations of a segment, and ε denoting an absolute residual error per sample.

Algorithm	OL	CS	POS	BB	EM	TCn	MCn	TCm ${}^{1}$	MCm ${}^{1}$
BU [14] ${}^{2}$	no	yes	yes	yes	SSR	$O(n^{2})$	O(n)	$O(m^{2})$	O(m)
SWAB [14]	yes	yes	yes	yes	SSR	$O(n^{2})$	O(n)	$O(m^{2})$	O(m)
mSWAB [12]	yes	yes	yes	yes	SSR	$O(n^{2})$	O(n)	$O(m^{2})$	O(m)
emSWAB [15]	yes	yes	yes	yes	SAD	$O(n^{2})$	O(n)	$O(m^{2})$	O(m)
PLAMLiS [18]	n/a	yes	yes	yes	ε	n/a	O(n)	$O(m^{2}\;log\;m)$	O(m)
PLAMLiS++ ${}^{3}$ [19]	n/a	yes	yes	yes	ε	n/a	O(n)	$O(m^{2})$	O(m)
SW [14] ${}^{2}$	yes	yes	yes	yes	SSR	O(n)	O(n)	$O(m^{2})$	O(m)
By Luo et al. [22]	yes	mixed	no	yes	ε	O(1)	O(n)	O(m)	O(m)
By Lemire [20]	(yes) ${}^{4}$	n/a	no	yes	SSR	O(1) ${}^{5}$	O(n)	O(m)	O(m)
SwiftSeg [21]	yes	no	no	(yes) ${}^{6}$	SSR, ε, ⋯	O(1)	O(1)	O(m)	O(1)
CPLR [16]	yes	yes	no	no	SSR	O(1)	O(1)	O(m)	O(1)
SF [17]	yes	yes	no	no	ε	O(1)	O(1)	O(m)	O(1)
fastSW (our contribution)	yes	yes	yes	no	SSR	O(1)	O(1)	O(m)	O(1)

^1^ In order to provide worst-case bounds, the buffer lengths or the maximum segment lengths are assumed to be as large as the dataset itself, respectively. This also provides for the highest achievable compression ratio. ^2^ The original source does not get obvious from the literature. ^3^ No name has been given to the algorithm, but it is an extension of PLAMLiS. ^4^ Although not explicitly stated in [20], the PLA algorithm of Lemire could be used for online processing extending its range sums for each new sample and calculating the slope fit and segment error with it. ^5^ Although the actual calculation of line fit and error happens in an O(1) step, it is based on a precalculated array of range sums for each sample of the sequence. ^6^ In general, SwiftSeg is based on a buffer. However, for the first order variant with segmentation and slope information, a buffer might not be necessary, or at least does not constrain the segment lengths.

**Table 2:** Instruction counts (IC) of 4D implementations of CPLR, SF, fastSW, and SW on an ARM Cortex-M4 microcontroller, with *n* denoting the length of the current segment.

Algorithm	min. IC	max. IC
CPLR	198	209
SF	252	420
fastSW	191	210
SW	53	161+n·35

## Data Availability

Our implementations can be obtained by mailing the first or the last author.

## Abbreviations

The following abbreviations are used in this manuscript:

AAD	Average Angular Deviation
BLE	Bluetooth Low Energy
BU	Bottom Up
CFG	Control Flow Graph
CPLR	Connected Piecewise Linear Regression
FPU	Floating Point Unit
IC	Instruction Count
ICR	Inverse Compression Ratio
ISA	Instruction Set Architecture
IMU	Inertial Measurement Unit
MoCap	Motion Capturing
PLA	Piecewise Linear Approximation
SF	Swing Filter
SLERP	Spherical Linear Interpolation
SSR	Sum of Squared Residuals
SW	Sliding Window
SWAB	Sliding Window and Bottom Up
WCET	Worst-Case Execution Time
